# A multilevel assessment of the social determinants associated with the late stage diagnosis of breast cancer

**DOI:** 10.1038/s41598-021-82047-0

**Published:** 2021-02-01

**Authors:** Nayara Priscila Dantas de Oliveira, Marianna de Camargo Cancela, Luís Felipe Leite Martins, Dyego Leandro Bezerra de Souza

**Affiliations:** 1 Postgraduate Programme in Collective Health, Federal University of Rio Grande do Norte–UFRN, Natal, RN Brazil; 2 Division of Surveillance and Analysis, Coordination of Prevention and Vigilance (CONPREV), Brazilian National Institute Cancer (INCA), Ministry of Health, Rio de Janeiro, RJ Brazil; 3 Division of Populational Research, Coordination of Prevention and Vigilance (CONPREV), Brazilian National Institute Cancer (INCA), Ministry of Health, Rio de Janeiro, RJ Brasil; 4 Department of Collective Health, Postgraduate Programme in Collective Health, Federal University of Rio Grande do Norte–UFRN, Natal, RN Brazil; 5 Research group on Methodology, Methods, Models and Outcomes of Health and Social Sciences (M3O), Faculty of Health Science and Welfare, Centre for Health and Social Care Research (CESS), University of Vic-Central University of Catalonia (UVic-UCC), Vic, Spain

**Keywords:** Cancer, Breast cancer, Cancer epidemiology, Cancer prevention

## Abstract

The advanced-stage diagnosis of breast cancer reveals the inequalities associated with socioeconomic conditions and the offer of health services. This study analyzes the prevalence of advanced breast cancer and its relationship with individual and contextual socioeconomic indicators and offer of health service. A cross-sectional study is presented herein, on the assessment of malignant breast neoplasms in women diagnosed between 2006 and 2015 (n = 195,201). Data were collected from the Hospital Cancer Registry (HCR), Atlas of Human Development in Brazil, and from the National Registry of Health Institutions (NRHI). A multilevel Poisson Regression was carried out with random intercept. The prevalence of advanced breast cancer diagnosis was 40.0%. Advanced staging was associated with younger age groups (PR 1.41), race/nonwhite (PR 1.13), lower education levels (PR 1.38), and public access to health services (PR 1.25). There was also an association with a low density of mammographic equipment (PR 1.08), and with low indices of local social inequality (PR 1.33) and human development (PR 0.80). This study maps and highlights the causes related to inequalities in the diagnosis of advanced breast cancer in Brazil, and presents essential data to reorient public policies and health-related actions to strengthen the control of breast cancer in Brazil.

## Introduction

Breast cancer has high incidence and mortality rates around the world. It is the most common type of cancer in women, with increasing incidence rates, independent from local socioeconomic development levels^1^.

Until 2040, approximately 980,000 new cancer cases are estimated in the world^2^. In Brazil, the estimate is 66,280 new breast cancer diagnoses per year for 2020–2022, with an estimated incidence rate of 61.6 new cases per 100,000 women. Breast cancer presents high incidence across all Brazilian geographic regions, with magnitudes two to three times higher than colon and rectal cancer^3^.

When detected early, breast cancer presents a high cure potential. However, advanced stage diagnosis is associated with worse prognosis, higher mortality risk, and less chances of surviving the disease^4^.

Studies indicated that developing countries (low- and middle-income countries) present flaws in the physical structure of health services, limited availability of technologies, and the slow diagnostic flows of public healthcare cause delays in the detection and assistance to breast cancer patients^5,6^. However, in high-income countries, with better-structured health services, the high incidence rates of breast cancer are accompanied by high indices of diagnosis at early stages. Women in developed countries undergo well-timed, adequate treatment, which affects breast cancer survival and mortality rates in these areas^5^.

In Brazil, despite all efforts of the public health system to improve the early detection of breast cancer, approximately 40% of women are diagnosed in advanced stages of the disease. The high prevalence of advanced stage diagnosis of breast cancer in Brazilian women is associated with the social vulnerability condition of the population^7^. Diagnostic delays also affect the Brazilian scenario, considered inversely proportional to the degree of organization of health systems^6^.

Diagnostic delays for breast cancer in developing countries can be related to the low offer and suboptimal territorial distribution of mammographic equipment^6,8^. This is due to limitations of financial resources of the health sector, which hinders mammographic exams and the offer of effective breast cancer treatment^6^.

Current scientific literature provides a wide discussion on the health-related inequalities related to early diagnosis and survival to breast cancer^7–10^. However, existing studies have not specifically analyzed the relationship between different hierarchic levels in the social determinants of advanced stage diagnosis in the Brazilian geographic regions.

The discussion on the social inequalities associated with the diagnosis of advanced breast cancer, considering the different hierarchic levels of the social determinants of health, enables a better comprehension of the impact of this chronic disease at individual and collective levels. This discussion is vital when elaborating funding strategies and reorienting crucial public policies for the control, prevention, and timely treatment of breast cancer^11,12^.

The objective of this study is to analyze the prevalence of advanced stage diagnosis of breast cancer and its relationship with individual and contextual socioeconomic indicators and health service offer in Brazil.

## Results

**Figure 1:**
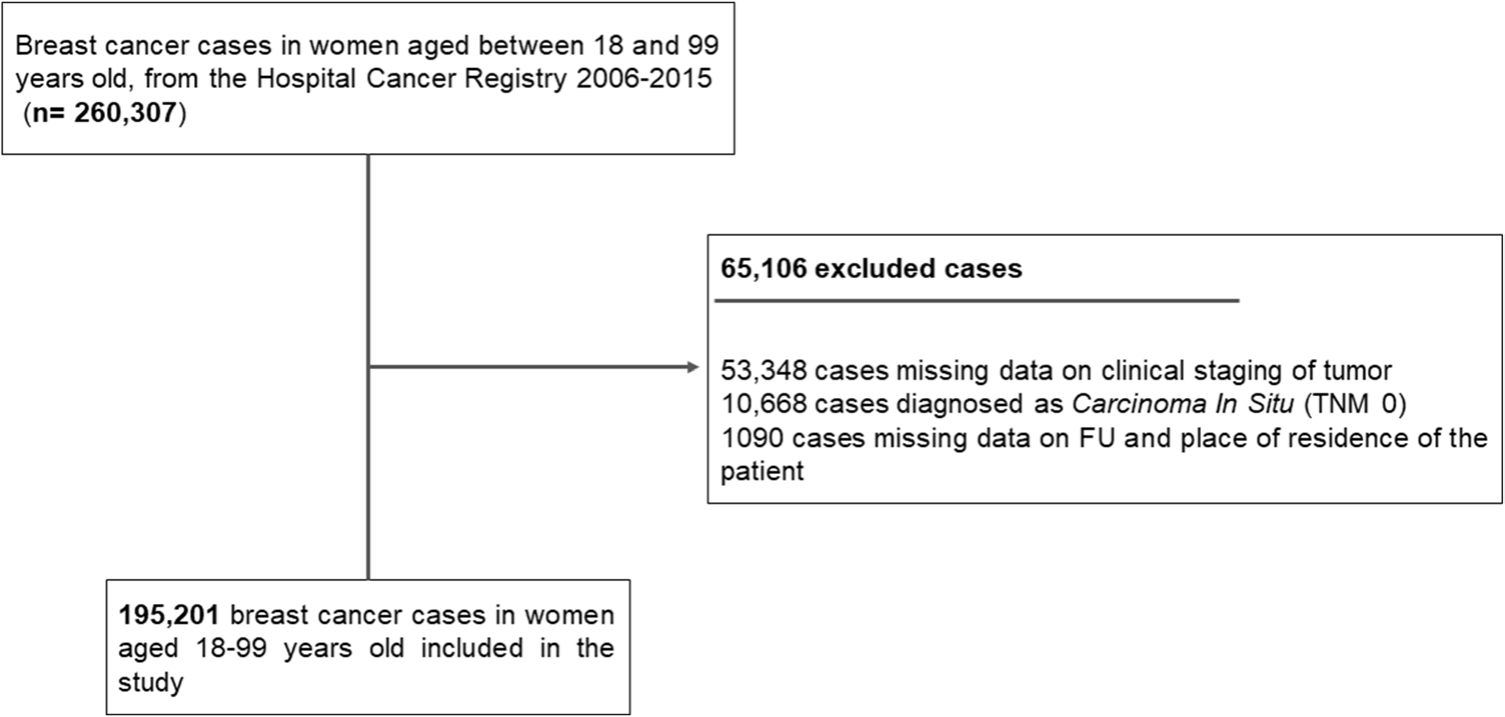
Flowchart of the selection process of the breast cancer cases, for 2006–2015, of the database of Hospital Cancer Registries.

Between 2006 and 2015, 260,307 breast cancer cases were registered in women between 18 and 99 years old. According to the eligibility criteria of this study, 195,201 breast cancer cases were included in the analysis, covering all FU of the country, as shown in Fig. 1.

**Figure 2:**
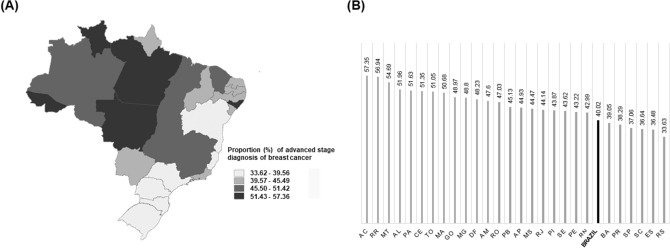
Spatial distribution of the proportion of advanced stage diagnosis of breast cancer (TNM III and IV) in Brazil, 2006–2015, per FU.

Figure 2 presents the spatial distribution of the proportions of advanced stage diagnosis of breast cancer for all 26 Brazilian FU and the Federal District. The percentage of advanced breast cancer staging in Brazil, for 2006–2015, was 40.0% (CI 39.8–40.2), varying across states and regions.

The highest proportions of advanced stage diagnosis of breast cancer are located in the North and Midwest regions, with emphasis on Acre (57.3%), Roraima (56.9%) and Mato Grosso (54.7%). For the Northeast region, Alagoas (52.0%) and Ceará (51.3%) are highlighted due to high proportions of advanced staging. The South and Southeast regions present moderate to low proportions of advanced stage diagnosis of breast cancer, with the highest percentages observed for Minas Gerais (48.8%) and Rio de Janeiro (44.1%), in the Southeast. The South presents lower proportions of advanced stage diagnosis of breast cancer, lower than the Brazilian average for the studied period.

For the bivariate and multilevel assessments, all breast cancer cases of the HCR of the state of São Paulo were excluded (n = 63,343). These analyses encompassed 131,858 breast cancer cases.

The prevalence and non-adjusted prevalence ratios (PR) for advanced breast cancer are presented in Table 1. Statistically significant associations are observed between advanced breast cancer staging and all individual variables related to biological factors (age group) and socioeconomic conditions (race-color, education levels, and type of access to health services). The same is observed for the associations between advanced breast cancer and the socioeconomic contextual variable Gini index, and indicators of the offer of health services to the population (density of gynecologists and density of mammographic equipment).

**Table 1:** Prevalence and non-adjusted prevalence ratios for advanced breast cancer staging, according to individual characteristics and contextual variables related to socioeconomic conditions and the offer of health services in Brazil, per FU, except São Paulo (n = 131,858).

	Advanced stage diagnosis of breast cancer				
	n	%	PR	CI (95%)	p*
**Individual variables**					
Age group					
18–29 years old	1016	55.9	1.37	1.28– 1.46	< 0.001*
30–39 years old	6511	50.4	1.24	1.20– 1.28	
40–49 years old	14,237	42.2	1.05	1.03– 1.07	
50–59 years old	14,278	40.0	1.00	–	
60–69 years old	10,010	37.3	0.94	0.91–0.96	
70 years old and over	8359	40.1	1.01	0.98–1.03	
Race/color					
Nonwhite	23,712	37.0	1.18	1.16–1.21	< 0.001*
White	26,493	46.0	1.00	–	
Education					
None/incomplete fundamental education	22,191	44.4	1.43	1.38–1.49	< 0.001*
Fundamental education	7954	40.8	1.33	1.28–1.39	
Secondary education/incomplete undergraduate education	9259	38.4	1.24	1.19–1.29	
Undergraduate education	3033	31.1	1.00	–	
Access to health services					
Public (SUS)	37,623	43.7	1.32	1.29–1.35	< 0.001*
Private/health plan	9316	35.0	1.00	–	
**Socioeconomic contextual variables**					
Gini index					
0.49–0.56	25,093	37.8	1.00	–	0.007*
0.57–0.60	9861	44.3	1.12	0.97–1.29	
0.61–0.65	19,457	45.1	1.18	1.06–1.31	
HDI					
0.63–0.68	24,164	44.8	1.12	0.97–1.30	0.232
0.69–0.74	17,936	38.2	1.05	0.90–1.22	
0.75–0.82	15,311	40.7	1.00	–	
**Health service offer contextual variables**					
Density of gynecologists					
2.25–12.70	20,694	44.7	1.15	1.01–1.32	0.008*
12.80–16.50	18,006	40.2	1.10	0.96–1.26	
16.60–48.63	15,711	38.5	1.00	–	
Density of operational mammographic equipment					
1.00–3.40	20,767	45.9	1.12	1.07–1.17	< 0.001*
3.50–4.60	18,298	37.8	1.02	0.98–1.05	
4.70–7.04	15,346	40.2	1.00	–	

*PR* prevalence ratio adjusted by the multilevel model with random intercept, *CI* confidence interval, *p* Wald’s test.

*Statistically significant.

Table 2 shows the results regarding the multilevel data assessment. The multilevel modeling, using the initial empty model, evidences that there is a statistical variation (different from zero) across Brazilian FU, according to the LR test (LR 804.27; p < 0.001).

**Table 2:** Multilevel analysis between individual, socioeconomic contextual, and health service offer-related variables and advanced breast cancer staging in women between 18 and 99 years old, in the period 2006–2015. Brazil, per FU, except SP (n = 131,858).

Variables	Empty model	Model 1		Model 2	
		PR (CI 95%)	p	PR (CI 95%))	p
**Level 1 (individual)**					
Age group					
18–29 years old	–	1.41 (1.32–1.50)	< 0.001	1.41 (1.32–1.50)	< 0.001*
30–39 years old	–	1.27 (1.23–1.31)		1.27 (1.23–1.31)	
40–49 years old	–	1.06 (1.04–1.09)		1.06 (1.04–1.09)	
50–59 years old	–	1.00		1.00	
60–69 years old	–	0.92 (0.92–0.95)		0.92 (0.90–0.95)	
70 years old and over	–	0.98 (0.96–1.01)		0.98 (0.96–1.01)	
Race/color					
Nonwhite	–	1.13 (1.11–1.15)	< 0.001	1.13 (1.10–1.15)	< 0.001
White	–	1.00		1.00	
Education					
None/incomplete fundamental education	–	1.38 (1.33–1.43)	< 0.001	1.38 (1.33–1.43)	< 0.001*
Fundamental education	–	1.27 (1.22–1.32)		1.27 (1.22–1.32)	
Secondary education/incomplete undergraduate education	–	1.16 (1.11–1.21)		1.16 (1.11–1.21)	
Undergraduate education	–	1.00		1.00	
Access to health services					
Public (SUS)	–	1.25 (1.22–1.28)	< 0.001	1.25 (1.22 1.28)	< 0.001
Private/health plan	–	1.00		1.00	
**Level 2 (aggregated by FU)**					
Gini index					
0.49–0.56	–	–	–	1.00	0.002*
0.57–0.60	–	–	–	1.15 (1.01–1.30)	
0.61–0.65	–	–	–	1.33 (1.14–1.56)	
HDI					
0.63–0.68	–	–	–	0.80 (0.68–0.93)	0.010
0.69–0.74	–	–	–	1.01 (0.91–1.13)	
0.75–0.82	–	–	–	1.00	
Density of gynecologists					
2.25–12.70	–	–	–	1.10 (0.98–1.23)	0.233*
12.80–16.50	–	–	–	1.08 (0.97–1.21)	
16.60–48.63	–	–	–	1.00	
Density of operational mammographic equipment					
1.00–3.40	–	–	–	1.08 (1.02–1.13)	0.001*
3.50–4.60	–	–	–	1.00 (0.97–1.03)	
4.70–7.04	–	–	–	1.00	
Fixed effects					
Intercept (CI 95%)	− 0.793 (− 0.85 to − 0.74)	0.266 (0.25 to 0.28)		0.223 (0.19 to 0.25)	
Random effects					
Variance (CI 95%)	0.018 (0.010–0.033)	0.014 (0.008–0.026)		0.0065 (0.003–0.013)	
LR test (x^2^. p-value)	804.27 (< 0.001)	559.98 (< 0.001)		186.88 (< 0.001)	

Model 1: Statistical model with inclusion of individual level variables; Model 2: Statistical model with inclusion of variables of individual level and contextual level per FU.

*PR* prevalence ratio adjusted by the multilevel model with random intercept, *CI* confidence interval, *p* Wald’s test.

*p trend ≤ 0.05.

The first model received the variables associated with individual characteristics. The results demonstrate the statistical significance of all presented variables and the fit between PR values and the respective confidence intervals (CI) with bivariate analysis. With the implementation of contextual variables (level 2), the statistical significance of all variables was maintained, except for “Density of gynecologists”. The fit of PR values was also identified. The variable “Density of gynecologists” was maintained in the model due to its theoretical plausibility and its fit with the statistical model.

For the final model, it was possible to identify a decline in variance (0.0065) in comparison with the initial empty model. Also, there was the maintenance of significance from the results of the LR (p < 0.001) test.

The results of the multilevel analysis indicate that advanced stage diagnosis of female breast cancer is associated with younger age groups (18 to 29 years old—RP 1.41; IC 1.32–1.50); with nonwhite race/color (RP 1.13; IC 1.10–1.15); with lower education levels (None or incomplete fundamental level—RP 1.38; IC 1.33–1.43); and with public access to cancer health services (RP 1.25; IC 1.22–1.28).

The advanced stage diagnosis of breast cancer was also significantly associated with a low density of operational mammographic equipment (RP 1.08; IC 1.02–1.13). Higher indices of local social inequality (RP 1.33; IC 1.14–1.56), and low HDI values (RP 0.80; IC 0.68–0.93) were significantly associated with advanced stage diagnosis of breast cancer. Nevertheless, the *p *trend test did not demonstrate statistical significance for HDI.

## Discussion

The results of the study indicate that advanced stage diagnosis of breast cancer is associated with individual factors as well as with characteristics of the social context in which women are inserted. The social vulnerability conditions, along with inequalities in the access to services and specific health care in Brazil, are related to advanced stage diagnosis of breast cancer. These findings evidence that young age, nonwhite race/color, and low education levels are determinant individual socioeconomic factors for the diagnosis of advanced female breast cancer. Besides, the associations identified with the public access to healthcare and the low density of mammographic equipment report the inequalities in the access to healthcare in Brazil.

In the sociopolitical context, the advanced stage diagnosis of breast cancer presented a positive association with high indices of local social inequalities and negative association with low levels of human development. This study identified a proportion of 40.0% advanced stage diagnosis of breast cancer throughout ten years, with regional variations that accompany the unequal distribution of resources and health-related technology in the Brazilian territory. The percentage of advanced stage diagnosis of breast cancer identified herein is compatible with other population-based studies developed in Brazil. The prevalence of the diagnosis of advanced female breast cancer has been reported as 40.2–53.5%^7,13^.

In countries with worse income distribution conditions, the proportion of women diagnosed at later stages of breast cancer is still higher than what was observed for Brazil. In Africa, the percentages of women diagnosed with breast cancer TNM III and IV vary between 46.0 and 71.0% in the North, and between 76.0 and 91.0% for Sub-Saharan Africa^14^. In more developed areas, the late detection of breast cancer presents lower prevalences, with decreasing trends throughout time. A study carried out in the USA identified a 30.0% prevalence of advanced breast cancer at diagnosis^15^. In Europe, data from Austria, Belgium, Germany, Italy, and Switzerland indicated a prevalence of 14.6% for advanced stage diagnosis of breast cancer between 2003 and 2012^16^.

The study presented herein postulates on the theory of SDH, starting from the principle that health is a complex social phenomenon^17^. The elevated prevalence of advanced stage diagnosis of breast cancer observed in developing countries highlights the health inequalities embedded in individual and collective socioeconomic conditions for the production of health-related outcomes.

Ethnical-racial, social, and economic inequalities related to the advanced stage diagnosis of breast cancer have been documented by several studies^7–9,13,15^. Despite the substantial reduction observed in these inequalities since the last decade, in emerging countries the levels of social and income inequalities are still significant^4^.

The diagnosis of advanced breast cancer was herein associated with the structural determinants of health that mark the social vulnerability conditions of the Brazilian female population. This finding corroborates with the profile of the women diagnosed with advanced stages of breast cancer by Santos-Silva et al. The results indicate that the diagnosis of advanced breast cancer was more prevalent in younger women, of black race/ethnicity, with low education levels, and that had to migrate to undergo cancer treatment^7^.

Advanced breast cancer can be the result of biological factors related to more aggressive, fast progression tumors (which are associated with younger age groups and black race/ethnicity, for example). Or a consequence of health inequalities that culminate in the diagnosis of advanced cancer, delays in assistance, and remote possibilities of treatment and cure^4^.

Women that live in socioeconomic deprivation are diagnosed with advanced breast cancer and have fewer chances of accessing diagnostic technologies and undergoing treatment^18^. Besides, the low socioeconomic position determines the exposure of women to a situation of social vulnerability, where access to primary conditions of life and healthcare is limited^18,19^.

This study exposes an association between advanced stage diagnosis of breast cancer and public access to cancer healthcare. In Brazil, the majority of the population uses the publicly funded health system to receive healthcare—which is universal and aims at integral and equal care^12^. The disproportionality between the high demand for specific healthcare and the low offer of services and technologies leads to the incapacity of the public system to assist the population^20^.

The fragility identified in the assistance to women with breast cancer culminates in diagnostic and assistance-related delays that can result in the search for private healthcare by a share of the population (approximately 25.0%)^20,21^. The remainder of the female population suffers with limited or even complete lack of access to health services, resources, and technology, which reflects on the outcomes associated with breast cancer in Brazil^11,13,21^.

A study developed in the USA demonstrated that women covered by private health plans had better access to healthcare, regarding preventive services and cancer treatment^10^. Data from a cross-sectional Brazilian national study evaluated the access to early detection services for breast cancer and revealed that the coverage of breast cancer preventive tests is higher in the female Brazilian population that uses private health services^22^.

Besides the difficult access to services, the issue of offer and availability of health-related equipment and technology must be discussed. The specialized services that present adequate infrastructure and trained health professionals for cancer assistance are usually located in the larger Brazilian urban centers. These facilities are commonly overburdened with the high demands for healthcare^23^.

The offer and access to health services in Brazil have been increasing in recent years, but the regional differences remain^24,25^. The South and Southeast regions present the best urban structures, with structured health services that are organized and distributed across the territory. The North and Northeast regions present irregular population occupations, which limits the distribution of health services in the territory. The Northeast region, in turn, concentrates technologies and health services in large urban centers, which limits the offer of intermediate-level healthcare^26,27^.

The use of mammographies to screen for breast cancer is considered an important secondary prevention strategy, aimed at the early detection of the neoplasm in asymptomatic phases, and acts as an intervention measure^5^. In Brazil, mammographic screening is recommended every 2 years for women aged 50–69 years old. However, the Brazilian breast cancer tracking program presents several fragilities and limitations, which prevent the analysis of the national coverage of this strategy^28,29^.

Brazilian studies describe an irregular distribution of mammographic equipment, both for public and private facilities, in the different regions of the country. The results indicate a concentration of technology in more developed urban areas, which hinders the access to mammographic tests in poorer and less developed regions^30,31^. It must be emphasized that the coverage of mammographic equipment is unequal in Brazil, with an offer of health services that is lower than the real necessities of the population^30^.

In developed countries, breast cancer monitoring programs are considered effective and cover an ample share of the population, employing scientific methods with high technological quality. Considering the North-American reality, 57.2% of women between 50 and 75 years old have regular screening mammographies^32^. Regarding the offer of mammographic equipment, Canada presents a density of 72 units per 1,000,000 inhabitants, which leads to high effectiveness and coverage of breast cancer screening programs^33^.

The collective socioeconomic and political context, herein represented by local social inequality indicators and human development indices, plays a vital role in the structure of SDH. The contextual aspects can help increase or decrease the effects of social inequalities on health-related outcomes and the wellness of individuals and their collectivities^17^.

The association established between the advanced stage diagnosis of breast cancer and higher levels of social and income inequalities (evaluated by the Gini Index) evidences the influence of context e on social stratification and health opportunities of the population. The Gini Index presents a significant trend, which was not detected for HDI, which reveals the higher impact of local social inequalities on the advanced stage diagnosis of breast cancer, and reinforces the vulnerability situation of populations.

The decrease in social and income inequalities observed in Brazil in recent decades has not occurred homogeneously across the country^34^. The advanced stage diagnosis of breast cancer presents a strong relationship with social and income disparities, as observed in the interior areas of the Brazilian FU. This is explained by the unequal distribution of resources and health assistance in the interior of the administrative units of the country^34,35^.

Regarding the HDI, it was not possible to observe a dose–response effect when these indicators were compared throughout the Brazilian FU in the context of breast cancer diagnosis. The results evidence that socioeconomic differences across FU (evaluated by the HDI) do not interfere with the social determinants of advanced stage diagnosis of breast cancer in Brazil. In opposition to these findings, existing scientific literature exposes an inverse relationship between HDI and advanced breast cancer diagnosis. In less developed countries and regions, TNM III and IV staging present higher detection rates^5,13^.

The utilization of secondary data sources can be a limiting factor in the study. However, HCR is the most complete source of secondary data related to cancer diagnosis in Brazil. The reliability of the results presented herein is ensured by the adoption of measures to control and adjust the variables.

The results of the study conclude that the advanced stage diagnosis of female breast cancer presents high prevalence rates and critical regional differences across the Brazilian territory. Individual and contextual factors help increase the health-related inequalities associated with advanced stage diagnosis of breast cancer in the female population. Inter-sector public policies should prioritize equity in the context of early detection of breast cancer in all Brazilian geographic regions. Actions directed to the control and secondary prevention of breast cancer should focus on the more vulnerable female populations, filling the existing health gaps in the social hierarchy. Public policies must follow the realities of each FU, considering the local social inequalities and offer of health services.

## Methods

### Study design and participants

This is an observational, cross-sectional study developed with secondary data extracted from the Hospital Cancer Registry (HCR)^36^. HCR gathers standardized information on the sociodemographic characteristics of cancer patients, clinical characteristics of the tumor, and hospital assistance activity^37,38^.

The analysis included cases of malignant neoplasms of the breast (C50)^39^ in women aged between 18 and 99 years old, assisted by hospital units for cancer treatment, diagnosed in 2006–2015.

Cases with missing data on TNM tumor staging were excluded, along with in situ (TNM 0) carcinoma cases and situations with no data regarding age and place of residence at the time of diagnosis.

Data from the HCR of the state of São Paulo were not included in the analysis due to its different data collection processes, with no individual information available on the socioeconomic conditions of cancer patients. Including this HCR would prevent data comparison.

### Variables

The outcome analyzed herein is advanced clinical tumor staging, classified by the TNM classification of malignant tumors^40^. Clinical staging of the primary tumor was dichotomized into advanced (TNM III and IV) and early (TNM I and II) stages*.*

**Figure 3:**
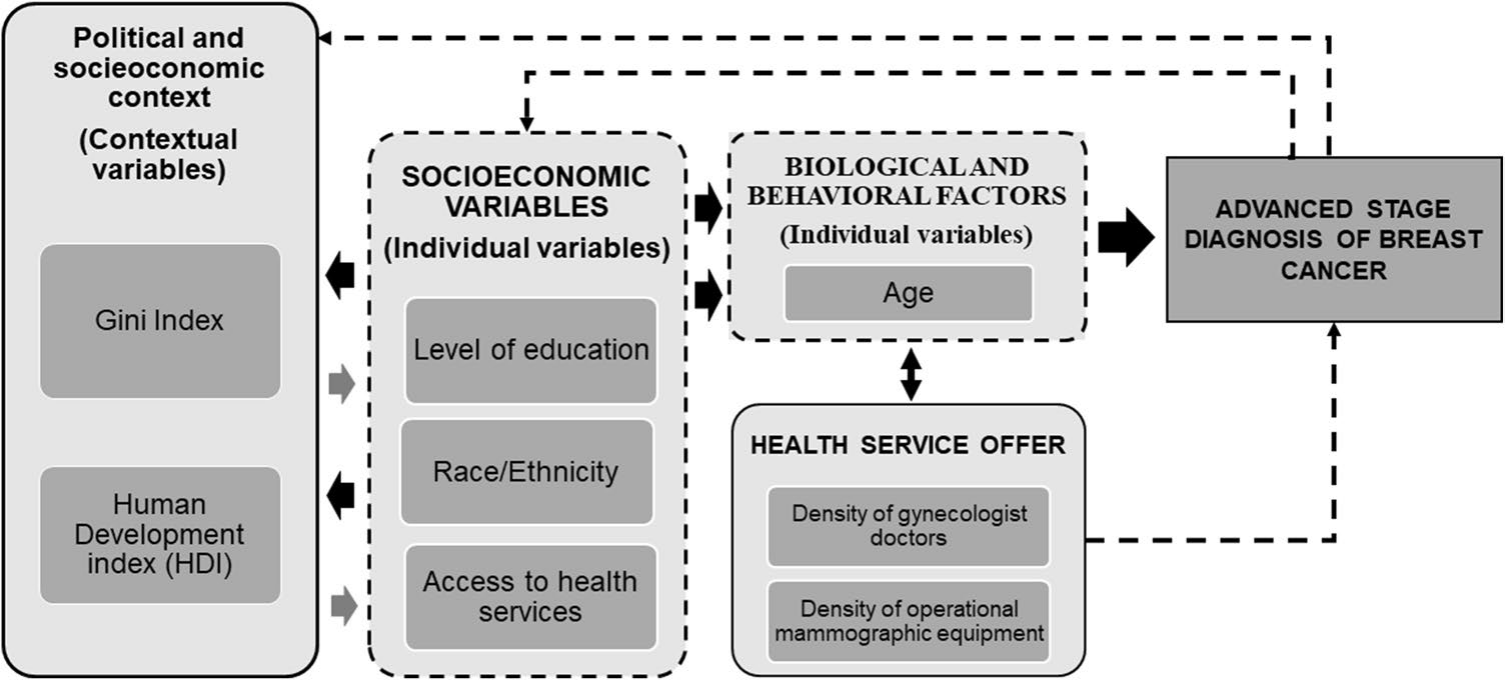
Theoretical model of the Social Determinants of Health related to advanced stage diagnosis of breast cancer in Brazil.

The independent variables (Fig. 3) are presented based on the theoretical conceptual model of the Social Determinants of Health (SDH) developed by the Commission of the Social Determinants of Health (CSDH) of the World Health Organization (WHO)^17^. This conceptual model approaches three essential components of SDH: sociopolitical context, structural determinants (socioeconomic position), and intermediate determinants of health^17^.

The sociopolitical context included the variables related to the contextual socioeconomic conditions of the Brazilian states (Gini index and Human Development Index, HDI). Structural determinants grouped the individual variables related to the socioeconomic position of women diagnosed with breast cancer (education level, race, and type of access to health services). Intermediate determinants encompassed biological and behavioral factors, along with local contextual indicators associated with the offer and access to health services (density of medical professionals and rate of availability of specific health equipment).

### Data sources

The individual databases were associated with two other databases with aggregated data per Federation Unit (FU): (a) data collected from the Atlas of Human Development in Brazil, made available by the United Nations Development Programme (UNDP)^41^, and (b) data on medical density and offer of health services, extracted from the National Registry of Health Institutions (NRHI)^42^. From these data, specific indicators were calculated for 2008 and 2013: “Density of Gynecologist Doctors” (number of gynecologist doctors per 100,000 women) and “Density of Mammographic equipment” (number of mammographic equipment per 100,000 women).

The Brazilian demographic census of 2010 was employed for the calculation of indicators, and population data and estimations per FU, sex, and age were carried out and reported by the Brazilian Institute of Geography and Statistics (IBGE)^43^. Quantitative variables were categorized in tertiles or as dichotomized variables (categorization by the median), when required by bivariate and multilevel data assessment.

### Statistical assessment

The first step was the descriptive analysis of data with a summary of measurements, tabulation, and the construction of graphics. The mechanisms of missing data of the HCR were previously analyzed^7^, which enables a complete analysis of cases herein.

The maps were elaborated from territorial geographic mesh (shape files), publicly available from the IBGE. The geographic meshes are made available in: https://www.ibge.gov.br/geociencias/organizacao-do-territorio/malhas-territoriais.html.

Spatial data analysis was carried out by georeferencing, with software TerraView 4.0.0, utilizing the FU to create specific maps. This analysis shows the spatial distribution of advanced stage diagnosis of breast cancer across the Brazilian territory between 2006 and 2015.

Pearson’s chi-squared test was applied to verify the association of the dependent variable with the independent variables of the study. Due to the characteristic of the outcome (prevalence higher than 10%) and utilization of contextual variables, the multivariate analysis strategy used a Multilevel Poisson regression, with random intercept, defined following the results of the Likelihood Ratio (LR) test.

Firstly, an empty model was analyzed, only with random intercept. Individual level variables were included, with random intercept—the reduction in the variability of the random effect was examined by comparing with the previous model. Then, contextual level variables were included in the modeling. The statistically significant variables were maintained in the model, according to Wald’s test (α = 0.05), along with those variables that presented theoretical plausibility for being included in the final statistical model. Individual and cross-level interactions were also tested^44^.

The p trend test was carried out to determine the dose–response effect between the independent variables of the study and the prevalence of advanced stage diagnosis of breast cancer, including the variables that constitute the final multilevel model^45^. All analyses were developed with Software Stata 15.1^3^.

The assessment of a Research Ethics Committee was not necessary for this study, according to Resolution 580/2018, because the secondary data employed were available from health information systems, publicly available, and it was not possible to identify the individuals^46^.
